# Early linear growth retardation: results of a prospective study of Zambian infants

**DOI:** 10.1186/s12889-019-6411-3

**Published:** 2019-01-14

**Authors:** Roma Chilengi, Mah Asombang, Jillian L. Kadota, Obvious N. Chilyabanyama, Katayi Mwila-Kazimbaya, Harriet Ng’ombe, Michelo Simuyandi, Samuel Bosomprah

**Affiliations:** 1 Research Division, Centre for Infectious Disease Research in Zambia, Plot # 34620, Off Alick Nkhata Road, PO Box 34681, Lusaka, Zambia; 2 Department of Biostatistics, School of Public Health, University of Ghana, Legon Accra, Ghana

**Keywords:** Growth retardation, Linear g*rowth*, *Growth velocity*, *Growth trajectory*, *Children*, *Prospective study*

## Abstract

**Background:**

Linear growth retardation is the most dominant nutritional problem globally. We aimed to describe linear growth trajectory among infants under 2 years of age using the WHO growth velocity standards.

**Method:**

This was a prospective cohort study of infants enrolled at 6 weeks of age and followed up for up to 24 months in Kamwala Urban Health Centre, Lusaka, Zambia. The study was conducted between April 2013 and March 2015. Infants were enrolled if they were 6–12 weeks of age and the mother was willing to participate voluntarily and provided informed consent. Anthropometric data were collected at scheduled clinic visits at 1 month, 2 months, 3 months, then quarterly until the infant was 24 months old. We defined linear growth velocity as the rate of change in height. We estimated linear growth velocity as the first derivative of the penalized cubic spline mixed effects model.

**Results:**

A total of 338 children were included in the analysis. Of these, 185 (54.7%) were female, 115 (34.1%) were born to HIV positive mothers and thus classified as HIV Exposed (HE). The mean age of children at enrollment was 1.6 months (SD = 0.15). On average, the growth velocity for 3-month length increments conditional on age were 0–3 months = 2.97 cm/3mo (95%CI = 2.69, 3.25); 3–6 months = 2.62 cm/3mo (95%CI = 2.38, 2.87); 6–9 months = 1.57 cm/3mo (95%CI = 1.43, 1.71); 9–12 months = 1.18 cm/3mo (95%CI = 1.08, 1.28); 12–15 month = 1.14 cm/3mo (95%CI = 1.02, 1.27); 15–18 months = 0.87 cm/3mo (95%CI = 0.79, 0.96); 18–21 months = 0.80 cm/3mo (95%CI = 0.72, 0.89); and 21–24 months = 0.86 cm/3mo (95%CI = 0.77, 0.96). For both boys and girls, the growth velocity in our cohort were consistently below the 3rd percentile of the WHO linear growth velocity standard. The estimated mean height and the age at which growth begins to falter were 68.6 cm (95%CI = 68.0, 69.2) and 13.6 months (95%CI = 13.2, 14.1) respectively.

**Conclusion:**

We found slower rate of growth among otherwise healthy Zambian infants. The data suggests that growth retardation is universal and profound in this cohort and may have already been occurring in utero.

## Background

Linear growth retardation is the most dominant nutritional problem globally, with approximately 22.2% or 151 million children under five reported to be stunted in 2017 [1]. Early Linear growth retardation has been shown to have long-term consequences and adversely affect adult stature and cognitive development [2, 3]. Attained growth for age, calculated as a child’s height-for-age z-score (HAZ), has been used as a measure of linear growth retardation. But this has limitations. Growth progresses at a rapidly decelerating rate from birth, reaching a near-plateau by the end of the first year, and continues to taper off gently through the second year. This is the expected trajectory of growth under conditions of adequate nutrition and psychosocial care. However, when there is a growth problem possibly due to inadequate nutrition and infections, the trajectory may change but attained growth for age is not robust enough to picking up early growth problems along this trajectory.

For this reason, we argue in this study that the human growth dynamic is complex and requires robust and clinically relevant indicators to monitor an individual’s growth trajectory. Several authors including Tanner JM 1952; Guo et al. 1991; and Roche & Himes 1980, have suggested that growth velocity can identify growth problems earlier than would have been if using attained growth [4–6]. They further indicated that pathogenic factors can directly affect velocity, while its effect on attained growth can be detected only after the results of growth velocity have manifested. In other words, height velocity quantifies the status of an infant at a fixed period whereas attained height indicates the results of what has happened in the past. For example, if a child has attained a height within the 50th percentile at 1-year of age, but does not grow for 1 year, at age 2 the child’s HAZ would be considered in the normal range. In contrast, his height velocity would show that there has been a stunted abnormal growth during the last 1 year. As evidenced by this example, height velocity is thus a dynamic measure, while attained growth measures are static. It is for these reasons that we considered growth velocity measure to be a more valuable tool and a superior measure for assessing infant growth compared to attained growth for age. Importantly, such growth velocity measures can lend to early identification of growth faltering, therefore making it possible to intervene promptly to avoid a reduced adult stature and related cognitive problems.

Despite this advantage however, very few studies have investigated infant growth using velocity measures. In this study, we aimed to describe the rate of growth among children under two years using the World Health Organization (WHO) growth velocity standards (WHO 2009) [7]. We also evaluated growth trajectory characteristics by determining the peak height and age at which growth begins to falter. Overall, we hope that findings from this study could provide insight into the usefulness of velocity measures as a dynamic and robust alternative for detecting early growth problems for improved care.

## Methods

### Study site and population

The study was undertaken at Kamwala Health Centre, a typical peri-urban clinic in Lusaka. The facility serves a large mixed population with a birth cohort more than 1400 deliveries each year. Within the premises of the clinic is the research facility, through which all under 5 clinic services were offered to the study participants. Recruitment was done while mothers come for the routine clinic visits; those with age eligible infants were invited to information sharing about the study. Motivated mothers were then invited to the research unit where informed consent was administered.

### Study design and participants

This was a prospective cohort study of infants conducted between April 2013 and March 2015. Details of the design were described elsewhere [8]. Children were enrolled if, i) they were 6–12 weeks of age, the mother was willing to participate voluntarily and able to provide signed informed consent (with witness in the case of illiterate participant; ii) the infant was eligible for rotavirus vaccine (RV) immunization as per national policy (male or female infant, aged 6–12 weeks old); iii) the mother was willing to undergo study procedures, including questionnaires, human immunodeficiency virus (HIV) counselling and testing, cluster of differentiation 4 (CD4) testing and provide breast milk at enrolment; iv) the mother was willing to allow her infant to undergo study procedures, including full course RV1 vaccination, phlebotomy at enrolment and 1 month post full RV2 vaccination, and presentation to clinic for collection of stool sample when infant had diarrhoea; and v) the mother planned to remain in the area and was willing to come for scheduled visits for the duration of the study. At enrolment, socio-demographic and anthropometric data were recorded. We also collected data on mother’s HIV status. The antenatal cards were inspected to verify the HIV status of the mother. Further, anthropometric data were collected at scheduled clinic visits at 1 month, 2 months, 3 months, then quarterly until the infant was 24 months old; thus, each child was expected to attend 10 scheduled visits. Mothers were also counselled to bring back the infant when ill; such visits constituted unscheduled clinic visits. Self-reports on any diarrhoea since the previous visit were also recorded.

### Statistical analysis

Summary statistics (mean and proportions) were used to describe participant’s baseline characteristics. This included infant age at first visit, gender, birthweight, mother’s age at infant birth, maternal HIV status at infant birth, and HAZ. We calculated HAZ for each baseline height measurement available for each child using the WHO child growth standards [WHO 2006] [9]. Stunting and severe stunting were defined as a HAZ < − 2 and HAZ < − 3, respectively. Based on these cut-offs, we calculated the proportion of children who were stunted and severely stunted at enrollment. We defined linear growth velocity as the rate of change in height. We included children who had at least three height measurements in the analysis.

We calculated growth velocity using two approaches. First, for each child, we calculated empirical growth velocity as the difference between two successive length or height measurements divided by the corresponding age gap. Using these values, we calculated the average 3-monthly age-specific growth velocity and 95% confidence interval (CI). The actual ages at which measurements were made were at times delayed (or advanced) compared to the target ages based on scheduled visits. This resulted in some measurement intervals being either longer or shorter than 91 days for a 3-month interval. For this reason, we corrected the actual measurement age to target age using maximum tolerable difference [7]. We adjusted standard errors for clustering of measurement within each child. The velocity and 95%CI for each age gap were presented using range plots for the entire sample and by sex. Second, we estimated linear growth velocity as the first derivative of the penalized cubic spline mixed effects model [10, 11]. This model has the functional form:

$$ {y}_{ij}=\left({b}_0+{\beta}_{0i}\right)+\left({b}_1+{\beta}_{1i}\right)x+\left({b}_2+{\beta}_{2i}\right){x}^2+\left({b}_3+{\beta}_{3i}\right){x}^3+\sum \limits_{k=1}^K\left({\mu}_k+{v}_{ki}\right)I\left(x>{K}_k\right){\left(x-{K}_k\right)}^3+{e}_{ij} $$

where $\left({\beta}_{0i},\dots, {\beta}_{3i}\right)\sim N\left(0,\Sigma \right),{\mu}_k\sim N\left(0,{\sigma}_{\mu}^2\right),{v}_{ki}\sim N\left(0,{\sigma}_v^2\right),$ Σ is an unstructured covariance matrix, K is the total number of knots at locations x = *K*_1_, *K*_2_, … , *K*_*k*_, and *I*(*x* > *K*_*k*_) is an indicator function that has value 1 if the condition *x* > *K*_*k*_ is true and 0 otherwise. For fitting *μ*_*k*_, we defined a variable that takes a value 1 to represent the whole sample as one super subject. In instances with readings that showed negative linear growth, we recoded as “no growth” by assigning the nominal value of + 0.01 to enable its inclusion in the statistical model.

We checked model adequacy using a plot of standardized residual against age. Having estimated the model, we obtained predicted height (based on both the fixed and random effects) for each child at each age. We then estimated the growth velocity as the numerical first derivative of the predicted height with respect to age. To describe the growth trajectory characteristics in terms of the peak height and age at which growth begins to decline, we identified for each child the age with the minimum absolute first derivative where the second derivative was negative. Infants who had less than three height measurements were excluded from the analysis due to the inability to calculate the rate of change of height over the duration of the study. The age and the height at that age were summarized to describe the trajectory characteristics. All analyses were performed using Stata 15 MP (StataCorp, College Station, TX, USA).

## Results

### Characteristics of participants

A total of 338 children were included in the analysis. Of these, 185 (54.7%) were female, 115 (34.1%) were born to HIV positive mothers and thus classified as HIV Exposed, and 222 (65.9%) were classified as HIV Unexposed. The mean age of children at enrollment was 1.6 months (SD = 0.15). At enrolment, the prevalence of stunting was 58.6% and severe stunting was 35.8% (Table 1).

**Table 1:** Baseline cohort characteristics

Characteristics of participants	*N* = 338
Child age at first visit (months), Mean (SD)	1.6 (0.15)
Child gender (female), n (%)	185 (54.7)
Child weight at first visit (kg), Mean (SD)	4.4 (1.1)
Mother’s age at birth (years), Mean (SD)	25.7 (5.7)
Maternal HIV status, n (%)	
Positive	115 (34.1)
Negative	222 (65.9)
Diarrheal episodes reported, Mean (SD)	1.1 (1.2)
Stunted at first measurement (<−2 SD), n (%)	198 (58.6)
Severely stunted at first measurement (< −3 SD), n (%)	121 (35.8)

### Characteristics of linear growth trajectory

**Fig. 1:**
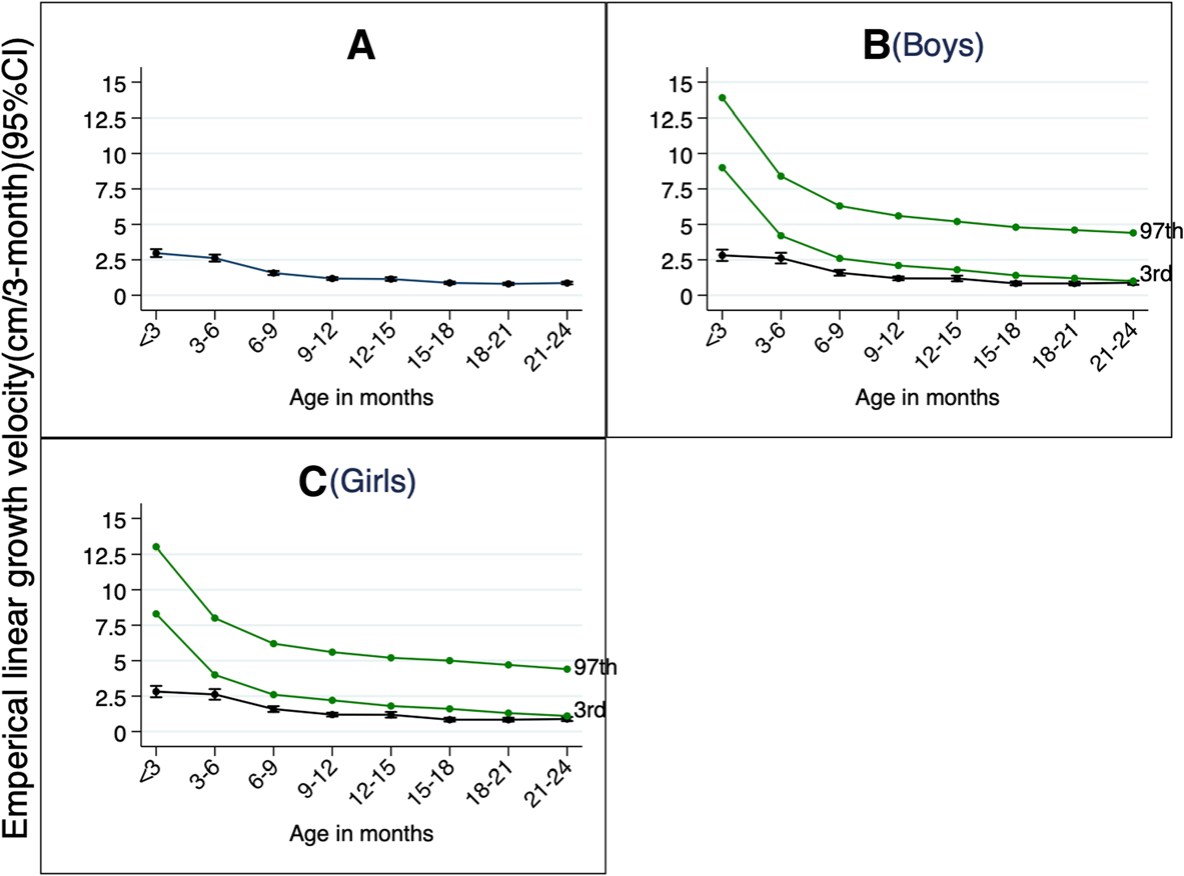
Three-monthly empirical linear growth velocity. Legend: In *green* are the WHO linear growth velocity standard (3rd and 97th percentiles). Overall growth velocity average per 3 months’ time (**a**); Average growth velocity per 3 months for the boys (**b**); Average growth velocity per 3 months for the girls (**c**)

On average, the growth velocity for 3-month length increments conditional on age were 0–3 months = 2.97 cm/3mo (95%CI = 2.69, 3.25); 3–6 months = 2.62 cm/3mo (95%CI = 2.38, 2.87); 6–9 months = 1.57 cm/3mo (95%CI = 1.43, 1.71); 9–12 months = 1.18 cm/3mo (95%CI = 1.08, 1.28); 12–15 month = 1.14 cm/3mo (95%CI = 1.02, 1.27); 15–18 months = 0.87 cm/3mo (95%CI = 0.79, 0.96); 18–21 months = 0.80 cm/3mo (95%CI = 0.72, 0.89); and 21–24 months = 0.86 cm/3mo (95%CI = 0.77, 0.96) (Fig. 1). For both boys and girls, the growth velocity in our cohort were consistently below the 3rd percentile of the WHO linear growth velocity standard (Fig. 1), suggesting that growth retardation is universal and profound in our cohort.

**Fig. 2:**
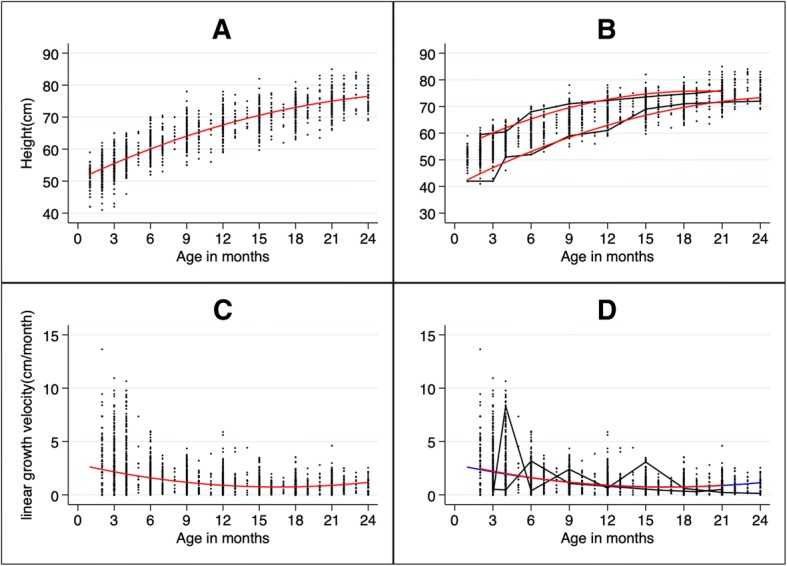
Observed and predicted linear growth using penalized cubic spline mixed effects model. Legend: In *black* are the observed growth curves and in *red* and *blue* are predicted growth curves. Observed and predicted linear growth curves for all children (**a**); for two individual children at different percentiles (**b**). Observed and predicted linear growth velocity for all children (**c**); for two individual children at different percentiles (**d**). The same children were plotted in B and D. Notice children have a different growth pattern but similar growth velocity. The knots were at 3, 4, 5, 6, 12, and 18 months

A visualization of individual growth of two children at two different percentiles and their predicted values showed our model effectively reflects individual and population growth patterns (Fig. 2b). Differences between children appeared to be largely from shifts in intercepts (Fig. 2b), with minimal differences in growth rates. This is further supported in the height velocity model in which the two individuals have almost the same rate of growth (Fig. 2d). Therefore, our model was able to effectively predict both population growth and differences between and within individuals in a population of Zambian children from a peri-urban shanty town. The estimated mean height and the age at which growth began to falter were 68.6 cm (95%CI = 68.0, 69.2) and 13.6 months (95%CI = 13.2, 14.1) (Fig. 2a). This is further supported by the near flat curve after age 13 months in Fig. 2c.

## Discussion

Our data showed that infant’s rate of growth were consistently below the 3rd percentile of the WHO growth velocity standards, implying inadequate nutrition even in the earlier periods of infancy. While the Zambian infants were growing at a slower rate, even by the time they entered the cohort at 6 weeks of age, the results from the present study also indicated that linear growth faltering is worst at 13.6 months of age. This is the time when nearly all the infants have completely been weaned off breastmilk.

A significant number of infants still do not benefit from optimum breastfeeding practices. Issaka et al report that in Southern Africa the overall prevalence of predominant breast feeding ranged between a lowest of 17.63% (95% CI 12.70 to 22.55) in East Africa and a highest of 46.37% (95% CI 37.22 to 55.52) in West Africa [12]. This is below the WHO/UNICEF optimum recommendation of breastfeeding for two years [1]. Breastfeeding for the first six months is crucial to child development and fundamental to the protection against illnesses. It gives infants all the nutrients they need for healthy development and contains antibodies that help protect infants from common childhood illnesses like diarrhea, and pneumonia: the two primary causes of childhood mortality worldwide [13].

While exclusive breastfeeding seems like a logical low/no cost intervention, its practice in Zambia is rather low. In our earlier study, the prevalence of exclusive breast feeding in children up to 5 months ranged between 39 and 45% [14]; and these findings are similar to what Tembo et al reported elsewhere, with a high start at 96% in the first two months of life and rapidly falling to 16% by 5 months [15]. Studies, including meta-analyses have demonstrated significant benefits of exclusive breastfeeding on diarrheal and pneumonia morbidity and mortality in children [16–18]. In early infant life, there are three issues of major concern for growth: first is whether the child was born healthy without any genetically determined defects; second, is the environment and hygiene practices to safeguard against infections; and third, of relevance to this paper is nutrition that the child needs to develop. The infants in our cohort were clinically healthy and enrolled during routine immunization visits, and thus somewhat normal growth trajectory was expected. However, our findings suggest, in the very least, that their nutrition may have been inadequate.

Inadequate nutrition not only predispose to acute morbidity (particularly diarrhea and pneumonia), it causes deleterious effects to infant’s growth, eventual cognitive development and future livelihood [17–19]. Thus, current global wisdom suggests that new born infants should be exclusively breastfed for the first six months of life and then additional soft feeds introduced gradually while breast feeding should continue for up to 18–21 months of age [20–22]. Early introduction to solid foods is problematic as it results in low iron stores by displacing energy rich and highly bioavailable iron in breastmilk, and increase the risk of diarrheal diseases [20, 23].

In our study, all mothers self-reported exclusive breast feeding, but it is likely that there could have been over reporting, especially given WHO global reports on Zambian that only 40% of infants less than six months of age are exclusively breastfed [24]. We have previously reported gross non-compliance to the international code for marketing breastmilk supplements by the private sector; and this very likely adds to negative influences against best breastfeeding practices in Lusaka [25]. However, the downside to exclusive breastfeeding is its association with increased risk of HIV transmission from mother to child [18, 26, 27]. This is particularly important in many areas of sub-Saharan Africa like Zambia where HIV prevalence is high [28].

However, the practicality of avoiding exclusive breastfeeding remains challenging even in light of maternal HIV especially among low social economic status populations. Cost, adequacy, hygiene, water safety and ability to appropriately mix formula are well known challenges [24, 29]. Indeed a relationship has been demonstrated between poor feeding practices and incidence of diarrhea in infants [30]. One episode of acute severe gastroenteritis is known to rob the child of substantial stored nutrients such as zinc and vitamins [31, 32]. Nutrient deficiencies can rarely occur in isolation to a single micronutrient, thus it is reasonable to expect that range of key growth nutrients (including copper, iron, magnesium, selenium, zinc, vitamins A, B_12_, D and folate) are lost together during diarrhea. Moreover, gastroenteritis often induces both vomiting and/or loss of appetite, and thus no replenishment is occurring during that period; therefore, it is logical to suggest that diarrhea deprives the child of both stored nutrition and food intake: material needed for growth [33, 34]. Our study reported an average of 1.1 (SD = 1.2) episodes of diarrhea per child over the duration of follow up, suggesting that each child experienced at least one episode of diarrhea between the time they enroll and exited the study.

While this study has showed a slower growth in our cohort, it had some limitations. First, it was not primarily designed as an anthropometric study; rather, the parent study was focused on understanding factors that influence rotavirus vaccine taken in healthy infants [8]. Second, although common to all longitudinal studies which follow up participants over time, some children did not have anthropometric data at all time points to be included in the statistical model; fortunately, this problem was limited because there is no evidence that the missingness were systematic to cause substantial bias. Also, children in our cohort did not have length measurement recorded at birth, which may have underestimated the empirical height velocity at 0–3 mo age group. Notwithstanding these limitations, there is a clear indication that the infant population under 2 years of age possesses a growth trajectory characteristic implying that they are growing slower than expected. Indeed, while others have shown that the HAZ approach may not be entirely accurate in showing actual height gain differences [35], these data have also highlighted the importance of growth velocity as a more robust and dynamic measure of linear growth.

## Conclusion

We found slower rate of growth among otherwise healthy Zambian infants. The data suggests that growth retardation is universal and profound in this cohort and may have already been occurring in utero. Growth retardation is a complex issue, which requires a robust and dynamic measures to detect early growth retardation among infants for improved and targeted care. Further research is needed to find best approaches to implement nutritional recommendations for the first 1000 days post-conception to prevent early growth faltering in Zambian infants.

## Abbreviations

CD4: Cluster of differentiation 4
CI: Confidence interval
CIDRZ: Centre for infectious diseases research in Zambia
HAZ: Height-for-age z-score
HIV: Human immunodeficiency virus
RV: Rotavirus vaccine
WHO: World Health Organization
